# Tumour suppressor microRNA-584 directly targets oncogene *Rock-1* and decreases invasion ability in human clear cell renal cell carcinoma

**DOI:** 10.1038/sj.bjc.6606028

**Published:** 2010-11-30

**Authors:** K Ueno, H Hirata, V Shahryari, Y Chen, M S Zaman, K Singh, Z L Tabatabai, Y Hinoda, R Dahiya

**Affiliations:** 1 Department of Urology, San Francisco Veterans Affairs Medical Center, University of California, San Francisco, 4150 Clement Street, San Francisco, CA 94121, USA; 2 Department of Pathology, San Francisco Veterans Affairs Medical Center, University of California, San Francisco, San Francisco, CA, USA; 3 Department of Oncology and Laboratory Medicine, Yamaguchi University Graduate School of Medicine, Yamaguchi, Japan

**Keywords:** miRNA-584, motility, RCC, ROCK-1

## Abstract

**Background:**

The purpose of this study was to identify new tumour suppressor microRNAs (miRs) in clear cell renal cell carcinoma (ccRCC), carry out functional analysis of their suppressive role and identify their specific target genes.

**Methods:**

To explore suppressor miRs in RCC, miR microarray and real-time PCR were performed using HK-2 and A-498 cells. Cell viability, invasion and wound healing assays were carried out for functional analysis after miR transfection. To determine target genes of miR, we used messenger RNA (mRNA) microarray and target scan algorithms to identify target oncogenes. A 3′UTR luciferase assay was also performed. Protein expression of target genes in ccRCC tissues was confirmed by immunohistochemistry and was compared with miR-584 expression in ccRCC tissues.

**Results:**

Expression of miR-584 in RCC (A-498 and 769-P) cells was downregulated compared with HK-2 cells. Transfection of miR-584 dramatically decreased cell motility. The ROCK-1 mRNA was inhibited by miR-584 and predicted to be target gene. The miR-584 decreased 3′UTR luciferase activity of ROCK-1 and ROCK-1 protein expression. Low expression of miR-584 in ccRCC tissues was correlated with high expression of ROCK-1 protein. The knockdown of ROCK-1 by siRNA inhibited cell motility.

**Conclusion:**

miR-584 is a new tumour suppressor miR in ccRCC and inhibits cell motility through downregulation of ROCK-1.

Renal cell carcinoma (RCC) is the tenth leading cause of death, accounting for 2–3% of adult malignancies (Jemal *et al*, 2008). The 5-year survival of RCC is estimated to be approximately 55% (Pascual and Borque, 2008) and that of metastatic RCC is approximately 10% (Reeves and Liu, 2009). Recently a multikinase inhibitor has been approved for the treatment of advanced RCC (Stadler *et al*, 2010), but is not globally used. Therefore, new sensitive, reliable tumour markers and effective therapeutic methods are needed for renal cancer. The mouse genome has been estimated to harbour 22 000 genes with 181 047 transcripts, however, protein-coding transcripts account for just 16 247 of the total (Carninci *et al*, 2005). These data show that non-coding RNAs are very numerous and potentially important in gene and protein regulation. These small non-coding RNAs and microRNA (miR) are well known as examples of non-coding RNAs (Ying *et al*, 2008). The miRs initially bind to the 3′UTR of target gene messenger RNA (mRNA) and repress translation or induce mRNA cleavage (McManus and Sharp, 2002), thereby inhibiting translation from mRNA to protein. Human miRs have been predicted to number approximately 1000 (Berezikov *et al*, 2005). Aberrant expression of miRs occurs in many types of cancers, some of which function as tumour suppressor genes or oncogenes (Lu *et al*, 2005; Esquela-Kerscher and Slack, 2006; Volinia *et al*, 2006). Decreased expression of tumour suppressor miRs results in increased expression of target oncogenes. In contrast, increased expression of oncogenic miRs leads to loss or decreased expression of target tumour suppressor genes. According to previous reports, a number of microRNA microarray studies have been performed in renal cancer patient samples and the expression level of several miRs (miR-17-5p, miR-20a, miR-21, miR-34a, miR-10a, miR-106b, miR-155 and miR-210) were validated by real-time (RT)–PCR and found to be upregulated in renal cancer tissues (Gottardo *et al*, 2007; Nakada *et al*, 2008; Huang *et al*, 2009; Jung *et al*, 2009; Petillo *et al*, 2009; Chow *et al*, 2010; Juan *et al*, 2010; Slaby *et al*, 2010; Weng *et al*, 2010). Some miRs (miR-141 and miR-200c) are downregulated in renal cancer compared with normal kidney tissues (Slaby *et al*, 2010). However, there have been few reports regarding the detailed functional analysis of these miRs in RCC.

Therefore, the aim of this study was to identify new tumour suppressor miRs that influence renal cancer progression, to validate the function of these tumour suppressor miRs and also to identify their target oncogenes.

In this study, we performed miR microarray analysis and validated microarray data by RT-PCR. Among several potential tumour suppressor miRs, miR-584 was downregulated in kidney cancer cell lines and was consistent with the microarray data. Rock-1 was selected on the basis of the microarray analysis and a target scan algorithm as a cell motility-related gene because miR-584 overexpression significantly inhibited cell motility. Therefore, we observed that miR-584 decreased cell motility through inhibition of ROCK-1 in RCC cell lines and the expression of miR-584 was inversely correlated with that of ROCK-1 in ccRCC (clear cell renal cell carcinoma) tissues. These results suggest that miR-584 functions as a new tumour suppressor miR in RCC via ROCK-1 knockdown.

## Materials and methods

### Cell lines and cell cultures

A-498 and 769-P cells originated from clear renal cell adenocarcinoma, and we selected them as model RCC cells. The HK-2 cells are derived from normal kidney and were used as control cells. The miR-584 expression level in A-498 and 769-P cells was lower than HK-2 cells, which was consistent with the finding that miR-584 expression in tumour tissue samples was lower compared with normal samples. In addition, A-498 and 769-P cells were used for functional studies of miR-584. The HK-2 cells were cultured in keratinocyte-SFM (Gibco/Invitrogen, Carlsbad, CA, USA). A-498 and 769-P cells were cultured in RPMI-1640 medium supplemented with 10% fetal bovine serum.

### RNA extraction

MiR and total RNA were extracted from cell lines using a miRNeasy Mini Kit and a RNeasy Mini Kit (Qiagen, Valencia, CA, USA). The miRs from clinical samples were extracted using laser-capture-micro-dissection techniques with a miRNeasy FFPE Kit (Qiagen).

### Microarray (miR and mRNA microarray)

For microRNA microarray, total RNA was extracted from HK-2 and A-498 cells using a miRNeasy Mini Kit. The miR microarray analysis was carried out and analysed by LC Science, LLC (Houston, TX, USA). We decided on candidate miR-584 for further experiments on the basis of the LC science microarray data. In order to find potential target oncogenes of tumour suppressive miR-584, we used an mRNA microarray service (Phalanx Bio Inc., Palo Alto, CA, USA). Namely, renal cancer cells (A-498 cells) were transfected with negative control or miR-584. Total RNA was extracted (miR-NC and miR-584 over-expression) after 72 h and Phalanx Bio Inc. performed mRNA microarray using the extracted RNA. On the basis of the microarray data, we identified several target oncogenes.

### Clinical samples

In total, 14 pairs of RCC and their normal adjacent tissues in paraffin blocks were obtained from NDRI (six pairs) (Philadelphia, PA, USA) and the Pathology Department of the Veterans Affairs Medical Center at San Francisco (eight pairs). Informed consent was obtained from eight patients from the Veterans Affairs Medical Center at San Francisco. All cancer samples were clear cell carcinoma with a mean age of 64.6 years (range, 43–81 years). According to the pTNM classification, the patient stages were as follows; 1 (stage I), 9 (stage II) and 4 (stage III).

### Transfection

Pre-miR miR precursor (negative control/hsa-miR-584, Ambion, Carlsbad, CA, USA), siRNA (control/ROCK-1, Invitrogen) and co-transfection of Pre-miR miR Precursor/pmirGLO Dual-Luciferase miR Target Expression Vector (Promega, Madison, WI, USA) were transiently transfected into cells by Lipofectamine 2000 (Invitrogen).

### Cell viability assay

Viability of A-498 and 769-P cells was measured by the MTS (CellTiter 96 Aqueous One Solution Cell Proliferation Assay, Promega) assay 6 days after transfection of Pre-miR miR Precursor. Cell viability was determined by absorbance measurements at 490 nm using SpectraMAX 190 (Molecular Devices, Sunnyvale, CA, USA).

### Matrigel invasion assay

Matrigel (1 : 5; BD Biosciences, San Jose, CA, USA) was added to Transwell membrane filter inserts (8.0-*μ*m pore size; BD Biosciences) and incubated for 5 h at 37°C in a 5% CO_2_ tissue culture incubator. A-498 and 769-P cells transfected with Pre-miR, miR Precursor or control were harvested 24 h after transfection and re-suspended in serum-free MEM medium. Aliquots (2 × 10^4^ cells) of the prepared cell suspension were added into the upper chamber and the lower chamber was filled with 1 ml of media containing fetal bovine serum. Cells were incubated for 48 h at 37°C in a 5% CO_2_ tissue culture incubator. Invasive cells were stained with Hema 3 Stain Set (Fisher Scientific, Pittsburgh, PA, USA). Cells per three random fields of each of the membranes were counted with Nikon ECLIPSE TS100 (Nikon, Tokyo, Japan) at × 100 magnification.

### Wound healing assay

A-498 and 769-P cells were seeded to 6-well plates and transfected with Pre-miR miR Precursor or control. At 24 h after transfection, cells were transferred from 6-well plates to 24-well plates. After 24 h, a wound was formed by scraping the cells with a 200 *μ*l tip and washing twice with medium. We observed cells at 0, 5, 8 and 24 h after scraping and photographed the cells with a microscope (Nikon).

### Luciferase reporter assay

A pmirGLO Dual-Luciferase miR Target Expression Vector was used for 3′UTR luciferase assays (Promega). The cell motility-related target oncogenes of tumour suppressor miR-584 were selected on the basis of a target scan algorithm (microRNA org. (http://www.microrna.org/microrna/home.do)) and microarray data (Phalanx Bio Inc.). The primer sequences used were as follows: *Rock1* forward primer, 5′-**AAAC**TAGCGGCCGCTAGTtgCATTTTTGCCAAGCCATAt**T**-3′ *Rock1* reverse primer, 5′-**CTAGA**aTATGGCTTGGCAAAAATGcaACTAGCGGCCGCTA**GTTT**-3′. Bold shows PmeI (AAAC/GTTT) and XbaI (T/CTAGA) sites.

In a total amount of 20 *μ*l, 5 *μ*l of 100 *μ*M forward primer, 5 *μ*l of 100 *μ*M reverse primer, 2 *μ*l of 10 × Annealing Buffer (100 mM Tris-HCl, pH 7.5, 1 M NaCl, 10 mM EDTA) and 8 *μ*l water were added to a 200 *μ*l PCR tube and cooled to room temperature for 1 h after incubating in a thermal cycler at 95°C for 5 min. The annealed oligonucleotides were ligated into the *Pme*I- *Xba*I site of pmirGLO Dual-Luciferase miR Target Expression Vector. Colony direct PCR was performed for insert recognition using REDTaq (Sigma, St Louis, MO, USA). The primers used were as follows: forward primer, 5′-CGTGCTGGAACACGGTAAAA-3′ reverse primer, 5′-GCAGCCAACTCAGCTTCCTT-3′ PCR parameters for cycling were as follows: 94°C for 3 min, 30 cycles of PCR at 94°C for 30 s, 55°C for 30 s and 72°C for 20 s, 72°C for 10 min and 4°C for 10 min. The PCR product was digested with Not I (TaKaRa/Fisher Scientific). The size of the vectors containing oligonucleotide inserts was about 200 and 100 bp confirmed by electrophoresis after the *Not*I sequence was incorporated into primers. Vectors were sequenced directly by an outside vendor (MCLAB, South San Francisco, CA, USA). For 3′UTR luciferase assay, A-498 cells were co-transfected with Negative Control or hsa-miR-584 and pmirGLO Dual-Luciferase miRNA Target Expression Vectors using Lipofectamine 2000 (Invitrogen). Luciferase assay was performed using the Dual-Luciferase Reporter Assay System (Promega) at 48 h after transfection.

### Quantitative RT–PCR

Extracted total RNA was reverse-transcribed into single-stranded cDNA using a High Capacity cDNA Archive Kit (Applied Biosystems, Foster City, CA, USA) and a TaqMan microRNA reverse transcription kit (Applied Biosystems). The RT-PCR was performed using first-strand cDNA with TaqMan Fast Universal PCR Master Mix (Applied Biosystems). The assay numbers for the mRNA endogenous control (*β*-actin), target gene, miR endogenous control (RNU48) and target miRs were as follows: β-actin (Hs99999903_m1), ROCK-1 (Hs00178463_m1), RNU48 (001006) and miR-584 (001624). Quantitative PCR was performed on an Applied Biosystems Prism 7500 Fast Sequence Detection System (Applied Biosystems). Quantitative PCR parameters for cycling were as follows: 95°C for 20 s 40 cycles of PCR at 95°C for 3 s, and 60°C for 30 s. All reactions were done in a 10-*μ*l reaction volume in triplicate. The mRNA and miR expression level were determined using the 2^−Δ*C*_t_^ method.

### Western analysis

At 72 h after transfection, cells were washed in ice-cold PBS and added to RIPA lysis and extraction buffer (Fisher Scientific) containing Protease Inhibitor Cocktail I (Millipore, Billerica, MA, USA). Dishes were incubated for 15 min on ice and cells were collected with a cell lifter and rotated for 30 min at 4°C followed by centrifugation at 14 000 r.p.m. for 10 min at 4°C. Total protein was analysed by western blotting using primary antibodies, followed by anti-mouse and anti-rabbit IgG HRP-conjugated secondary antibodies (Cell Signaling Technology, Beverly, MA, USA), and were visualised with LumiGLO Reagent and peroxide reagent (Cell Signaling Technology). The primary antibodies used were anti-ROCK1 Antibody (BD Biosciences # 611136) and anti-beta-actin (Cell Signaling Technology #3700) antibodies.

### Immunohistochemistry (IHC)

In total, 14 paraffin-embedded specimens were used for IHC. Antigen retrieval was carried out by microwaving in citrate buffer (Thermo Scientific, Waltham, MA, USA). Slides were incubated overnight with anti-RCOK-1 antibody (# ab45171, Abcam, Cambridge, MA, USA). The Thermo Scientific Lab Vision Ultra Vision Detection System (Thermo Scientific) was used as a chromogen.

### Knockdown of ROCK-1 mRNA

Renal cancer cells were transfected with *ROCK-1* siRNAs (si-*ROCK-1* (HSS109292)), Invitrogen) or negative control siRNA (si-*NC*, Invitrogen) using Lipofectamine 2000 according to the manufacturer's instructions. The si*RNAs* sequences are as follows; *ROCK-1* siRNA sequence, 5′-UGAUCUUGUAGCUCCCGCAUGUGUC-3′.

### Statistical analysis

All statistical analyses were performed using GraphPad prism 5 software (GraphPad Software, San Diego, CA, USA). A *P*-value of <0.05 was regarded as statistically significant.

## Results

### Expression level of miR-584 in cell lines and primary tissues

**Figure 1:**
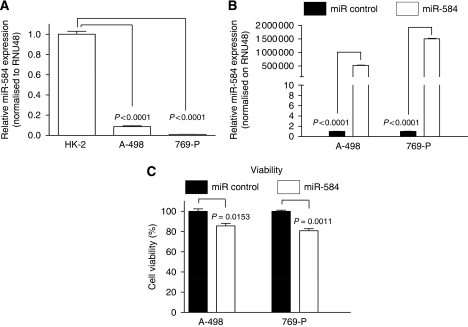
Expression of miR-584 in RCC cell lines and primary human tissues. (**A**) The HK-2 and RCC cell lines (A498 and 769-P). The miR-584 expression was normalised to RNU48. Data are presented as mean value±s.d. for three independent experiments and compared with the level of miR-584 in HK-2 cells normalised as 1. (**B**) MiR-584 expression levels were detected by using RT-PCR at 72 h after transfection. Data are presented as the mean value±s.d. for three independent experiments compared with the level of miR-584 obtained in miR control-transfected cells that is normalised to 1. (**C**) Cell viability was analysed by the MTS assay 6 days after transient transfection.

To identify tumour suppressor miR in RCC, we screened a miR microarray using normal kidney HK-2 cells and RCC A-498 cells. To confirm miR-584 expression, we performed RT-PCR and found that miR-584 expression in renal cancer cells (A-498 and 769-P) was significantly lower than that in HK-2 (Figure 1A). To analyse miR-584 expression in clinical samples, total RNA was extracted from 14 pairs of RCC and their adjacent normal FFPE tissues and RT-PCR was performed. As consistent with the results in cell lines, the miR-584 expression was significantly lower in renal cancer tissues (9/14, 64%) than in normal tissues (Table 1).

### Evaluation of functional effects of miR-584 on A-498 and 769-P cells

**Figure 2:**
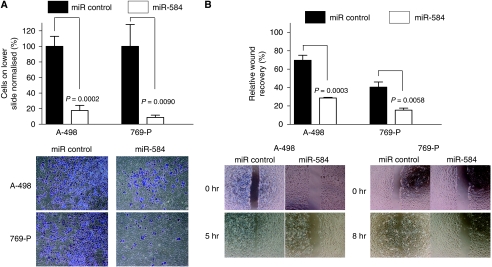
Evaluation of the functional effects of miR-584 in transfected A-498 and 769-P cells. (**A**) Effect of miR-584 transfection on cell invasion. At 48 h after transfection, a cell suspension was added into the upper chamber of matrigel-coated transwell membrane inserts and the lower chamber was filled with media and cultured for 48 h. Invasive cells were stained and the average number of cells was counted in triplicate. (**B**) Wound healing assay with miR-584-transfected cells. At 48 h after transfection, cells were transferred from 6-well to 24-well plates and further incubated for 24 h. A wound was formed by scraping and the wound measured after 5 h (A-498 cells) and 8 h (769-P cells).

To analyse the function of miR-584 in RCC, miR-584 and miR control were transiently transfected into A-498 and 769-P cells. The expression level of miR-584 was significantly increased at 72 h after transfection (Figure 1B). Cell viability was decreased in miR-584 transfectants compared with controls at 6 days after transfection (Figure 1C). Cell motility was also significantly decreased in miR-584-transfected renal cancer cells (Figure 2A and B).

### Evaluation of target genes of miR-584

**Figure 3:**
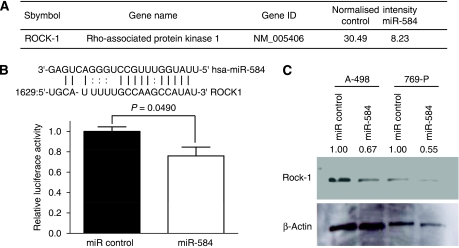
ROCK-1 is a miR-584 target gene. (**A**) Messenger RNA microarray data of ROCK-1. The miR-584 or miR control were transfected into A-498 cells. At 72 h after transfection, total RNA was extracted and mRNA expression level was compared by Phalanx Bio Inc. (**B**) Sequence of miR-584/ROCK-1 and 3′UTR luciferase assay. The miR-584 binding site in the ROCK-1 3′UTR predicted with microRNA org. (upper). 3′UTR vector and miR-584 or miR control were co-transfected into A-498 cells. Cell lysates were measured for relative luciferase activities at 48 h after transfection. Levels of luciferase activity were compared with those of miR control-transfected cells that is normalised to 1. (**C**) The ROCK-1 protein levels in RCC cells transfected with miR-584. The miR-584 or miR control was transfected into A-498 and 769-P cells. At 72 h after transfection, total protein was extracted and analysed by western blots. *β*-Actin was used as a loading control. The ratio of band intensity is relative to that of *β*-actin. Band intensity was measured by using ImageJ software.

To identify the target genes of miR-584, we initially used mRNA microarray to compare the expression level between miR-584-transfected A-498 cells and miR controls (data not shown). Second, we searched for target oncogenes of tumour suppressor miR-584 on the basis of the target scan algorithms (microRNA org. and TargetScanHuman 5.1) and selected the target oncogene, ROCK-1. To assay the inhibitory effect of miR-584 on ROCK-1 gene transcription, 3′UTR luciferase assay was performed with A-498 cells. The luciferase activity in miR-584-transfected cells was decreased to about 80% of that compared with miR control (Figure 3B). To examine the inhibitory effect of miR-584 on protein level, we did western analysis at 72 h after miR-584 transfection into A-498 and 769-P cells. We observed that protein levels of ROCK-1 in miR-584-transfected A-498 and 769-P cells were significantly decreased compared with miR control (Figure 3C).

### Relationship between tumour suppressor miR-584 expression and target oncogene ROCK-1 protein expression levels in renal cancer tissues

**Figure 4:**
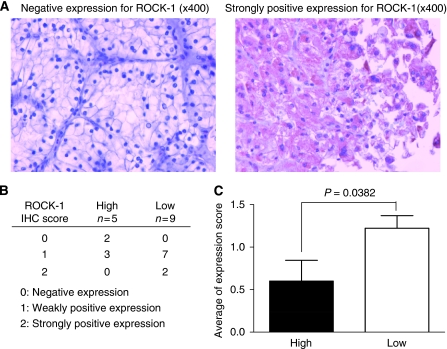
Expression of ROCK-1 in primary clear renal cancer tissues. (**A**) Representative immunostaining of ROCK-1 in primary tissue samples. Picture on the left shows negative expression, right shows strongly positive expression. (**B**) Summary of immunostaining scores. (**C**) The ROCK-1 expression in primary ccRCC tissues was compared between cases with higher miR-584 expression and those with lower miR-584 expression.

To compare the relationship between miR-584 and ROCK-1 expression in renal cancer tissues, the expression level of ROCK-1 protein in 14 primary ccRCC tissues were examined by IHC. Typical immunohistochemical findings of ROCK-1 are shown in Figure 4A. The expression level of ROCK-1 was assessed with an IHC score and compared with that of miR-584 (Figure 4B). The ROCK-1 protein expression was significantly higher in ccRCC cases with lower miR-584 expression than in those with higher miR-584 expression (Figure 4C).

### Evaluation of the effects of ROCK-1 knockdown on A-498 and 769-P cell motility

**Figure 5:**
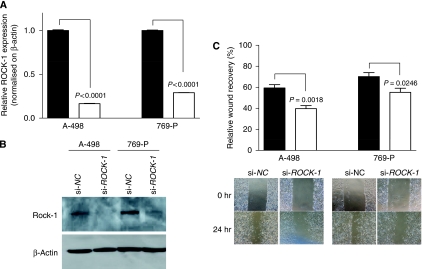
Functional effects of ROCK-1 knockdown on A-498 and 769-P cells. (**A**) Expression of ROCK-1 mRNA. si-ROCK-1 or si-NC were transfected into A-498 and 769-P cells. At 48 h after transfection, ROCK-1 mRNA was analysed using RT-PCR. ROCK-1 expression was normalised to *β*-actin. (**B**) ROCK-1 protein levels. At 72 h after transfection, total protein was extracted and analysed by western blots. *β*-actin was used as a loading control. (**C**) Wound healing assay of si-ROCK-1 transfectants. si-ROCK-1or si-*NC* was transfected into A-498 and 769-P cells. At 48 h after transfection, cells were transferred from 6-well to 12-well plates and further incubated for 24 h. A wound was then formed by scraping and measured after 24 h.

To analyse whether ROCK-1 affects cell motility of A-498 and 769-P cells, ROCK-1 siRNA (si-ROCK1) and control siRNA (si-NC) were transfected into A-498 and 769-P cells. The ROCK-1 mRNA was analysed using RT-PCR at 48 h after transfection. The ROCK-1 mRNA was significantly decreased in si-ROCK-1-transfected A-498 and 769-P cells (Figure 5A). Knockdown of ROCK-1 protein expression was also confirmed by western blot analysis (Figure 5B). To look at whether ROCK-1 knockdown affects cell motility, a wound healing assay was carried out using A-498 and 769-P cells. Cell motility was significantly decreased in si-ROCK-1 transfectants compared with control cells (Figure 5C).

## Discussion

There have been several miR studies related to renal cancer and these reports have initially dealt with miR profiling (Gottardo *et al*, 2007; Nakada *et al*, 2008; Huang *et al*, 2009; Jung *et al*, 2009; Petillo *et al*, 2009; Slaby *et al*, 2010; Weng *et al*, 2010). We also a used miR microarray service to compare miR expression levels between normal kidney cell line (HK-2) and renal cancer cell lines (A-498). On the basis of the miR microarray data (not shown), we chose miR-584 as a new potential tumour suppressor candidate miR as it was significantly downregulated in kidney cancer cells and there are no reports regarding the role of miR-584 in RCC. We also performed RT-PCR using HK-2 and A-498 cells and clinical samples (renal cancer and matched normal kidney tissues) to validate the microarray data and found that miR-584 expression level was significantly lower in renal cancer cell line (A-498) compared with normal kidney cell line (HK-2). As expected from the cell line data, miR-584 expression level was also significantly lower in primary ccRCC tissues than in matched normal kidney tissues (*n*=14). It has been reported that the expression level of miR-584 in malignant mesothelioma was higher compared with normal mesothelium, but functional analysis was not performed in that report (Guled *et al*, 2009). So far there have been no reports regarding miR-584 in other cancers, including renal cancer.

On the basis if these results and the previous report, we hypothesised that miR-584 may have an important role as a tumour suppressor in kidney cancer. To test this hypothesis, we performed functional analyses (MTS, invasion, migration, apoptosis, and cell cycle) to look at miR-584 function using miR-584-transfected cells. As expected, miR-584 over-expression inhibited cell proliferation in renal cancer cells (A-498 and 769-P). Cell invasion and motility were also dramatically decreased after miR-584 transfection. These results suggest that miR-584 may function as a tumour suppressor and have an important role in the inhibition of invasion and migration of renal cancer cells. Therefore, we looked for invasion-related genes as potential target oncogenes of miR-584. Initially, we did microarray profiling to compare mRNA expression between miR-584 transfectants and control cells. We also used microRNA org. to identify target oncogenes of miR-584. Among candidate target oncogenes, we found that ROCK-1 mRNA and protein expression were significantly downregulated in miR-584-transfected A-498 cells. To confirm specific binding of miR-584 to ROCK-1 mRNA, a 3′UTR luciferase assay was performed. These results show that luciferase activity was decreased after co-transfection of miR-584 and a 3′UTR vector containing the ROCK-1 miR-584 target sequence. These results show that ROCK-1 is a direct target of miR-584.

To validate whether miR-584 has a tumour suppressive role through ROCK-1 knockdown, we knocked down ROCK-1 in A-498 and 769-P cells using a si-RNA technique and did functional analysis with these cells. ROCK-1 mRNA and protein were downregulated in A-498 and 769-P cells after si-ROCK-1 transfection. To analyze cell motility, wound healing assay was carried out and cell motility was decreased in both A-498 and 769-P cells transfected with ROCK-1 siRNA. These results are the first to demonstrate that ROCK-1 regulates cell motility in RCC.

We also investigated the relationship between miR-584 and ROCK-1 expression levels in renal cancer tissues (*n*=14). We observed that ROCK-1 protein expression was significantly higher in ccRCC cases with low miR-584 expression than in those with high miR-584 expression. However, no association was found between miR-584 expression/ROCK-1 IHC score and clinico-pathological factors. Thus, our data show an inverse relationship between ROCK-1 and miR-584 expression levels.

It has been reported that ROCK-1 is over-expressed in brain tumour (Oellers *et al*, 2009) and related to metastasis in rat MM1 hepatoma cells, bladder, lung and prostate cancer (Itoh *et al*, 1999; Kamai *et al*, 2003; Chen *et al*, 2008; Lin *et al*, 2008). ROCK-1, which is activated by activated RhoA, promotes cell invasion and motility in prostate cancer and colorectal carcinoma cells (Wilkinson *et al*, 2005; Lin *et al*, 2008). Regarding the relationship between RhoA, B, C and Rock-1 in renal cancer, Abe *et al* (Abe *et al*, 2008) has reported that the RhoC and ROCK-1 mRNA expression levels are related to progression in ccRCC. RhoB, RhoC and ROCK-1 mRNA levels were significantly higher in ccRCC tissues compared with non-cancer tissues. However, RhoA mRNA expression was similar in ccRCC and non-cancer tissues (Abe *et al*, 2008). Although tumour grade and stage were related to RhoC and ROCK-1 in ccRCC, they were not related to RhoB. Expression of RhoC and ROCK-1 mRNA was highly correlated with High RhoC and ROCK-1 mRNA expression significantly associated with shorter survival in ccRCC (Kamai *et al*, 2002; Abe *et al*, 2008). Though these studies are interesting, the exact molecular relationship between RhoC and ROCK-1 in RCC is unclear because ROCK-1 is generally activated by RhoA (Fujisawa *et al*, 1996; Ishizaki *et al*, 1996).

Our data shows that the expression level of miR-584 is inversely correlated with that of ROCK-1. Given that the high expression level of ROCK-1 in RCC was shown to be associated with shorter survival (Abe *et al*, 2008), our present findings that ROCK-1 is a target gene of miR-584, the expression level of miR-584 may be correlated with survival and be involved in ccRCC progression.

In conclusion, this is the first report to show that miR-584 functions as a tumour suppressor, directly targets oncogene *ROCK-1* and decreases cell motility in RCC cells; we have also shown that miR-584 and ROCK-1 expressions are inversely correlated in primary ccRCC tissues. The limitation of this study is the small number of samples. Therefore, additional studies will be required to define the molecular mechanisms involved in the regulation of ROCK-1 by the Rho family.

## Figures and Tables

**Table 1:** The miR-584 expression and ROCK-1 IHC score in clinical sample

**No.**	**miR-584 expression level**	**ROCK-1 IHC score**	**Grade**	**pT**
1	2.7703	0	3	pT2
2	2.7218	1	2	pT1a
3	1.6948	1	1	pT1b
4	1.5945	0	2	pT1b
5	1.5578	1	2	pT2
6	0.7740	1	2	pT1b
7	0.6613	2	2	pT2
8	0.5520	2	3	pT1b
9	0.5352	1	2	pT1a
10	0.3918	1	2	pT2
11	0.2910	2	3	pT1a
12	0.1592	1	2	pT1a
13	0.1295	1	3	pT2
14	0.0671	1	2	pT1b

Abbreviations: IHC=immunohistochemistry; RCC=renal cell carcinoma.

The miR-584 expression level was compared between RCC and adjacent noncancerous (normal) tissues. The miR-584 expression was normalised to RNU48. The expression level in normal tissues is normalized as 1.

## References

[bib1] Abe H, Kamai T, Tsujii T, Nakamura F, Mashidori T, Mizuno T, Tanaka M, Tatsumiya K, Furuya N, Masuda A, Yamanishi T, Yoshida K (2008) Possible role of the RhoC/ROCK pathway in progression of clear cell renal cell carcinoma. Biomed Res 29: 155–161 18614849 10.2220/biomedres.29.155

[bib2] Berezikov E, Guryev V, van de Belt J, Wienholds E, Plasterk RH, Cuppen E (2005) Phylogenetic shadowing and computational identification of human microRNA genes. Cell 120: 21–24 15652478 10.1016/j.cell.2004.12.031

[bib3] Carninci P, Kasukawa T, Katayama S, Gough J, Frith MC, Maeda N, Oyama R, Ravasi T, Lenhard B, Wells C, Kodzius R, Shimokawa K, Bajic VB, Brenner SE, Batalov S, Forrest AR, Zavolan M, Davis MJ, Wilming LG, Aidinis V, Allen JE, Ambesi-Impiombato A, Apweiler R, Aturaliya RN, Bailey TL, Bansal M, Baxter L, Beisel KW, Bersano T, Bono H, Chalk AM, Chiu KP, Choudhary V, Christoffels A, Clutterbuck DR, Crowe ML, Dalla E, Dalrymple BP, de Bono B, Della Gatta G, di Bernardo D, Down T, Engstrom P, Fagiolini M, Faulkner G, Fletcher CF, Fukushima T, Furuno M, Futaki S, Gariboldi M, Georgii-Hemming P, Gingeras TR, Gojobori T, Green RE, Gustincich S, Harbers M, Hayashi Y, Hensch TK, Hirokawa N, Hill D, Huminiecki L, Iacono M, Ikeo K, Iwama A, Ishikawa T, Jakt M, Kanapin A, Katoh M, Kawasawa Y, Kelso J, Kitamura H, Kitano H, Kollias G, Krishnan SP, Kruger A, Kummerfeld SK, Kurochkin IV, Lareau LF, Lazarevic D, Lipovich L, Liu J, Liuni S, McWilliam S, Madan Babu M, Madera M, Marchionni L, Matsuda H, Matsuzawa S, Miki H, Mignone F, Miyake S, Morris K, Mottagui-Tabar S, Mulder N, Nakano N, Nakauchi H, Ng P, Nilsson R, Nishiguchi S, Nishikawa S, Nori F, Ohara O, Okazaki Y, Orlando V, Pang KC, Pavan WJ, Pavesi G, Pesole G, Petrovsky N, Piazza S, Reed J, Reid JF, Ring BZ, Ringwald M, Rost B, Ruan Y, Salzberg SL, Sandelin A, Schneider C, Schönbach C, Sekiguchi K, Semple CA, Seno S, Sessa L, Sheng Y, Shibata Y, Shimada H, Shimada K, Silva D, Sinclair B, Sperling S, Stupka E, Sugiura K, Sultana R, Takenaka Y, Taki K, Tammoja K, Tan SL, Tang S, Taylor MS, Tegner J, Teichmann SA, Ueda HR, van Nimwegen E, Verardo R, Wei CL, Yagi K, Yamanishi H, Zabarovsky E, Zhu S, Zimmer A, Hide W, Bult C, Grimmond SM, Teasdale RD, Liu ET, Brusic V, Quackenbush J, Wahlestedt C, Mattick JS, Hume DA, Kai C, Sasaki D, Tomaru Y, Fukuda S, Kanamori-Katayama M, Suzuki M, Aoki J, Arakawa T, Iida J, Imamura K, Itoh M, Kato T, Kawaji H, Kawagashira N, Kawashima T, Kojima M, Kondo S, Konno H, Nakano K, Ninomiya N, Nishio T, Okada M, Plessy C, Shibata K, Shiraki T, Suzuki S, Tagami M, Waki K, Watahiki A, Okamura-Oho Y, Suzuki H, Kawai J, Hayashizaki Y, FANTOM Consortium, RIKEN Genome Exploration Research Group and Genome Science Group (Genome Network Project Core Group (2005) The transcriptional landscape of the mammalian genome. Science 309: 1559–1563 16141072 10.1126/science.1112014

[bib4] Chen J, Ye L, Zhang L, Jiang WG (2008) Placenta growth factor, PLGF, influences the motility of lung cancer cells, the role of Rho associated kinase, Rock1. J Cell Biochem 105: 313–320 18615591 10.1002/jcb.21831

[bib5] Chow TF, Mankaruos M, Scorilas A, Youssef Y, Girgis A, Mossad S, Metias S, Rofael Y, Honey RJ, Stewart R, Pace KT, Yousef GM (2010) The miR-17-92 cluster is over expressed in and has an oncogenic effect on renal cell carcinoma. J Urol 183: 743–751 20022054 10.1016/j.juro.2009.09.086

[bib6] Esquela-Kerscher A, Slack FJ (2006) Oncomirs – microRNAs with a role in cancer. Nat Rev Cancer 6: 259–269 16557279 10.1038/nrc1840

[bib7] Fujisawa K, Fujita A, Ishizaki T, Saito Y, Narumiya S (1996) Identification of the Rho-binding domain of p160ROCK, a Rho-associated coiled-coil containing protein kinase. J Biol Chem 271: 23022–23028 8798490 10.1074/jbc.271.38.23022

[bib8] Gottardo F, Liu CG, Ferracin M, Calin GA, Fassan M, Bassi P, Sevignani C, Byrne D, Negrini M, Pagano F, Gomella LG, Croce CM, Baffa R (2007) Micro-RNA profiling in kidney and bladder cancers. Urol Oncol 25: 387–392 17826655 10.1016/j.urolonc.2007.01.019

[bib9] Guled M, Lahti L, Lindholm PM, Salmenkivi K, Bagwan I, Nicholson AG, Knuutila S (2009) CDKN2A, NF2, and JUN are dysregulated among other genes by miRNAs in malignant mesothelioma -A miRNA microarray analysis. Genes Chromosomes Cancer 48: 615–623 19396864 10.1002/gcc.20669

[bib10] Huang Y, Dai Y, Yang J, Chen T, Yin Y, Tang M, Hu C, Zhang L (2009) Microarray analysis of microRNA expression in renal clear cell carcinoma. Eur J Surg Oncol 35: 1119–1123 19443172 10.1016/j.ejso.2009.04.010

[bib11] Ishizaki T, Maekawa M, Fujisawa K, Okawa K, Iwamatsu A, Fujita A, Watanabe N, Saito Y, Kakizuka A, Morii N, Narumiya S (1996) The small GTP-binding protein Rho binds to and activates a 160 kDa Ser/Thr protein kinase homologous to myotonic dystrophy kinase. EMBO J 15: 1885–1893 8617235 PMC450107

[bib12] Itoh K, Yoshioka K, Akedo H, Uehata M, Ishizaki T, Narumiya S (1999) An essential part for Rho-associated kinase in the transcellular invasion of tumor cells. Nat Med 5: 221–225 9930872 10.1038/5587

[bib13] Jemal A, Siegel R, Ward E, Hao Y, Xu J, Murray T, Thun MJ (2008) Cancer statistics, 2008. CA Cancer J Clin 58: 71–96 18287387 10.3322/CA.2007.0010

[bib14] Juan D, Alexe G, Antes T, Liu H, Madabhushi A, Delisi C, Ganesan S, Bhanot G, Liou LS (2010) Identification of a microRNA panel for clear-cell kidney cancer. Urology 75: 835–841 20035975 10.1016/j.urology.2009.10.033

[bib15] Jung M, Mollenkopf HJ, Grimm C, Wagner I, Albrecht M, Waller T, Pilarsky C, Johannsen M, Stephan C, Lehrach H, Nietfeld W, Rudel T, Jung K, Kristiansen G (2009) MicroRNA profiling of clear cell renal cell cancer identifies a robust signature to define renal malignancy. J Cell Mol Med 13: 3918–3928 19228262 10.1111/j.1582-4934.2009.00705.x PMC4516539

[bib16] Kamai T, Arai K, Sumi S, Tsujii T, Honda M, Yamanishi T, Yoshida KI (2002) The rho/rho-kinase pathway is involved in the progression of testicular germ cell tumour. BJU Int 89: 449–453 11872041 10.1046/j.1464-4096.2001.01920.x

[bib17] Kamai T, Tsujii T, Arai K, Takagi K, Asami H, Ito Y, Oshima H (2003) Significant association of Rho/ROCK pathway with invasion and metastasis of bladder cancer. Clin Cancer Res 9: 2632–2641 12855641

[bib18] Lin SL, Chiang A, Chang D, Ying SY (2008) Loss of mir-146a function in hormone-refractory prostate cancer. RNA 14: 417–424 18174313 10.1261/rna.874808 PMC2248249

[bib19] Lu J, Getz G, Miska EA, Alvarez-Saavedra E, Lamb J, Peck D, Sweet-Cordero A, Ebert BL, Mak RH, Ferrando AA, Downing JR, Jacks T, Horvitz HR, Golub TR (2005) MicroRNA expression profiles classify human cancers. Nature 435: 834–838 15944708 10.1038/nature03702

[bib20] McManus MT, Sharp PA (2002) Gene silencing in mammals by small interfering RNAs. Nat Rev Genet 3: 737–747 12360232 10.1038/nrg908

[bib21] Nakada C, Matsuura K, Tsukamoto Y, Tanigawa M, Yoshimoto T, Narimatsu T, Nguyen LT, Hijiya N, Uchida T, Sato F, Mimata H, Seto M, Moriyama M (2008) Genome-wide microRNA expression profiling in renal cell carcinoma: significant down-regulation of miR-141 and miR-200c. J Pathol 216: 418–427 18925646 10.1002/path.2437

[bib22] Oellers P, Schröer U, Senner V, Paulus W, Thanos S (2009) ROCKs are expressed in brain tumors and are required for glioma-cell migration on myelinated axons. Glia 57: 499–509 18814230 10.1002/glia.20777

[bib23] Pascual D, Borque A (2008) Epidemiology of kidney cancer. Adv Urol 782381 19009036 10.1155/2008/782381 PMC2581742

[bib24] Petillo D, Kort EJ, Anema J, Furge KA, Yang XJ, Teh BT (2009) MicroRNA profiling of human kidney cancer subtypes. Int J Oncol 35: 109–114 19513557 10.3892/ijo_00000318

[bib25] Reeves DJ, Liu CY (2009) Treatment of metastatic renal cell carcinoma. Cancer Chemother Pharmacol 64: 11–25 19343348 10.1007/s00280-009-0983-z

[bib26] Slaby O, Jancovicova J, Lakomy R, Svoboda M, Poprach A, Fabian P, Kren L, Michalek J, Vyzula R (2010) Expression of miRNA-106b in conventional renal cell carcinoma is a potential marker for prediction of early metastasis after nephrectomy. J Exp Clin Cancer Res 29: 90 20609231 10.1186/1756-9966-29-90 PMC2907341

[bib27] Stadler WM, Figlin RA, McDermott DF, Dutcher JP, Knox JJ, Miller Jr WH, Hainsworth JD, Henderson CA, George JR, Hajdenberg J, Kindwall-Keller TL, Ernstoff MS, Drabkin HA, Curti BD, Chu L, Ryan CW, Hotte SJ, Xia C, Cupit L, Bukowski RM, ARCCS Study Investigators (2010) Safety and efficacy results of the advanced renal cell carcinoma sorafenib expanded access program in North America. Cancer 116: 1272–1280 20082451 10.1002/cncr.24864

[bib28] Volinia S, Calin GA, Liu CG, Ambs S, Cimmino A, Petrocca F, Visone R, Iorio M, Roldo C, Ferracin M, Prueitt RL, Yanaihara N, Lanza G, Scarpa A, Vecchione A, Negrini M, Harris CC, Croce CM (2006) A microRNA expression signature of human solid tumors defines cancer gene targets. Proc Natl Acad Sci USA 103: 2257–2261 16461460 10.1073/pnas.0510565103 PMC1413718

[bib29] Weng L, Wu X, Gao H, Mu B, Li X, Wang JH, Guo C, Jin JM, Chen Z, Covarrubias M, Yuan YC, Weiss LM, Wu H (2010) MicroRNA profiling of clear cell renal cell carcinoma by whole-genome small RNA deep sequencing of paired frozen and formalin-fixed, paraffin-embedded tissue specimens. J Pathol 222: 41–51 20593407 10.1002/path.2736

[bib30] Wilkinson S, Paterson HF, Marshall CJ (2005) Cdc42-MRCK and Rho-ROCK signalling cooperate in myosin phosphorylation and cell invasion. Nat Cell Biol 7: 255–261 15723050 10.1038/ncb1230

[bib31] Ying SY, Chang DC, Lin SL (2008) The microRNA (miRNA): overview of the RNA genes that modulate gene function. Mol Biotechnol 38: 257–268 17999201 10.1007/s12033-007-9013-8 PMC7091389

